# SmileGNN: Drug–Drug Interaction Prediction Based on the SMILES and Graph Neural Network

**DOI:** 10.3390/life12020319

**Published:** 2022-02-21

**Authors:** Xueting Han, Ruixia Xie, Xutao Li, Junyi Li

**Affiliations:** 1 School of Computer Science and Technology, Harbin Institute of Technology (Shenzhen), Shenzhen 518055, China; 21s051051@stu.hit.edu.cn (X.H.); lixutao@hit.edu.cn (X.L.); 2 School of Medical Technology and Nursing, Shenzhen Polytechnic, Shenzhen 518055, China; xieruixia07@szpt.edu.cn

**Keywords:** drug–drug interaction prediction, graph neural network, knowledge graph, structural features, topological features

## Abstract

Concurrent use of multiple drugs can lead to unexpected adverse drug reactions. The interaction between drugs can be confirmed by routine in vitro and clinical trials. However, it is difficult to test the drug–drug interactions widely and effectively before the drugs enter the market. Therefore, the prediction of drug–drug interactions has become one of the research priorities in the biomedical field. In recent years, researchers have been using deep learning to predict drug–drug interactions by exploiting drug structural features and graph theory, and have achieved a series of achievements. A drug–drug interaction prediction model SmileGNN is proposed in this paper, which can be characterized by aggregating the structural features of drugs constructed by SMILES data and the topological features of drugs in knowledge graphs obtained by graph neural networks. The experimental results show that the model proposed in this paper combines a variety of data sources and has a better prediction performance compared with existing prediction models of drug–drug interactions. Five out of the top ten predicted new drug–drug interactions are verified from the latest database, which proves the credibility of SmileGNN.

## 1. Introduction

Drug–drug interaction (DDI) prediction is one of the focuses of biomedical research. For many diseases with complex pathways of action, the use of a single drug may not be ideal for treatment. One solution is combination drug therapy, which uses several drugs at the same time. For instance, leukemia can be effectively treated by the concurrent use of Venetoclax and Idasanutlin, with Venetoclax inhibiting the anti-apoptotic Bcl-2 family protein and Idasanutlin activating the p53 pathway [1]. However, the concurrent use of multiple drugs may lead to adverse drug events (ADEs) [2,3]. Although DDIs can be confirmed by routine in vitro and clinical trials, it is difficult to test DDIs extensively and effectively before drugs are marketed. Due to the large number of drugs and the time cost of verification, it is almost impossible to test DDIs for every two drugs. At the same time, due to the fact that ADEs are not always reported and counted in time after the occurrence, there are relatively few documented and verified DDIs compared with the large number of drugs.

At present, DDI prediction methods are mainly divided into two categories: the drug structural feature-based approach and graph-based approach.

The drug structural feature-based approach assumes that chemically similar drugs have similar DDIs. Ryu et al. [4] proposed the DeepDDI model, which is the first model to use deep learning in drug–drug interaction prediction. Structural Similarity Profiles (SSP) of pairs of drugs are generated by using SMILES (Simplified Molecular Input Line Entry Specification) data of the drugs. PCA (Principal Components Analysis) is then used for dimension reduction. Finally, the SSPs are sent into the Deep Neural Network (DNN) for classification. On the basis of DeepDDI, Lee et al. [5] added two new data with the method similar to how SSP is generated by drugs’ SMILES data: target gene data to generate TSP (Target Similarity Profile) and gene ontology (GO) to generate GSP (Gene Ontology Term Similarity Profile). These three feature vectors (SSP, TSP, and GSP) are reduced in dimension by an improved encoder and then are stitched into a single feature vector for the drug pair, which is put into DNN for training. This improved model combines more data and has a higher accuracy. Based on the DeepDDI, a polymorphic deep learning model was proposed by Deng et al. [6], which uses the filtered complete information for training. It can use the information related to a variety of drugs to learn more efficiently and has a higher accuracy. The methods based on drug features have high accuracy on known data sets but they also have some limitations. The hypothesis that “drugs with similar chemical structures have similar DDIs” has not been scientifically verified. Thus, there may be a large deviation in the prediction results in actual clinical verification.

In recent years, a series of studies on the application of graph theory in the molecular level have achieved great success. Many researchers are trying to use graph theory for DDI prediction. Marinka et al. [7] proposed the model Decagon, which is a two-layer heterogeneous graph. It is constructed to predict the type of polypharmacy side effects of drug pairs whose drug targets are all proteins. In this study, the Graph Neural Network (GNN) is used to train the model by graph representation learning and it is shown that the GNN has better performance in predicting DDIs than both the traditional shallow graph structure model and the traditional graph embedding method. Bougiatiotis et al. [8] extracted the three-dimensional relationships related to a specific disease from various databases and expressed them with the Unified Medical Language System (UMLS) to construct multiple knowledge graphs (KG) for specific diseases. The model DDI-BLKG extracts drug features based on its pathways, which has a certain enlightenment for the prediction of DDIs. Lin et al. [9] extracted a large number of drug-related data from the database and processed data into triples. The triples were encoded to construct a huge KG. The feature vectors of drugs were generated through two times of aggregation by GNN. Thus, the vector includes not only the information of the drug itself but also the information of drug-related entities. The method based on graphs can model more drug data, such as the drug action pathway, and uses methods such as deep learning to make predictions. The graph-based method has a good explanatory power but sometimes neglects the information contained in the entities.

The Graph Neural Network (GNN) extends the convolutional neural network to non-Euclidean space, which provides a more natural and effective method for the modeling of graph structured data [10]. GNN can be regarded as an embedding method which extracts the embedding vectors of adjacent nodes for updating its own embedding vectors without the need for manual feature engineering [11]. In recent years, GNN has been widely used in the molecular level and has shown excellent performance [7,9,12,13].

The Knowledge Graph (KG), as a knowledge representation and management method, was proposed by Google in 2012. In recent years, KG has become popular in academia and industry, and its use has expanded from the search engine field to all fields involving big data [14]. The application of KG to DDI prediction also got good results [8,9,15,16,17]. KG is a kind of data structure based on graphs and is usually represented as triples, i.e., G = (head, relation, and tail). The head and tail are the head entity and tail entity, respectively, which are different entities generated from web pages. Relation is the relation in the knowledge base, which is transformed from the hyperlink of the web page into the semantic relation between entities.

## 2. Methodology

### 2.1. Drug Structural Features

**Figure 1:**
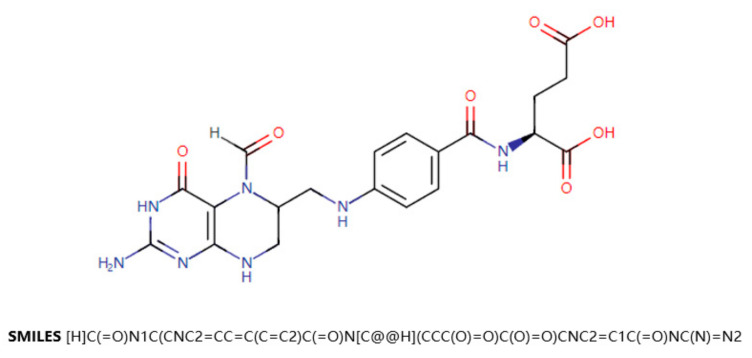
Two-dimensional graphs of the drug Leucovorin and its corresponding SMILES.

One of the main data sources for this paper is DrugBank [18]. DrugBank is a drug knowledge database that describes clinical information on drugs, such as side effects, DDIs, etc. DrugBank also provides data on the molecular level, such as the chemical structure of the drug, the target protein of the drug, etc. SMILES (Simplified Molecular Input Line Entry Specification) is a specification that explicitly describes molecular structures using ASCII strings. SMILES can describe a three-dimensional chemical structure with a string of characters. For example, Figure 1 shows a two-dimensional graph of the drug Leucovorin and its corresponding SMILES. SMILES can be imported by molecular editing software and converted into two-dimensional graphics or three-dimensional models of molecules.

**Figure 2:**
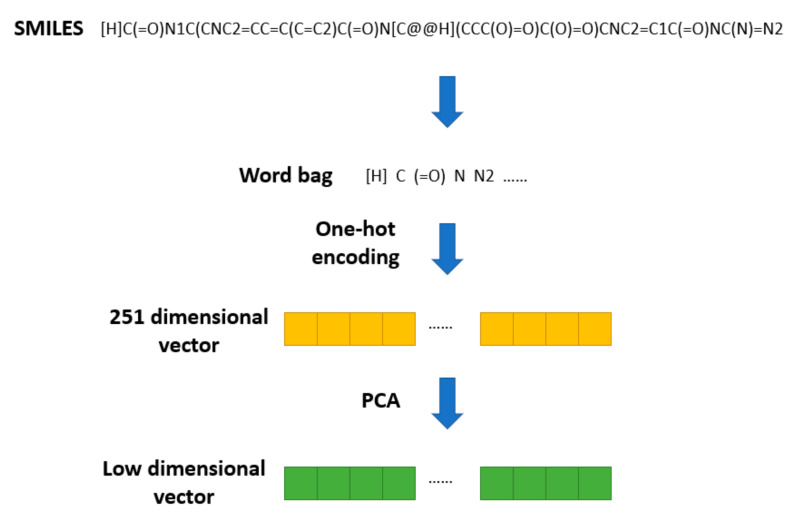
Pretreatment methods of SMILES.

The SMILES2Vec [19] method was proposed to apply Seq2seq [20] technology in natural language processing to a SMILES string. In SMILES2Vec, chemical structure information is used as an input variable into the deep neural network to predict the physical properties of compounds. SMILES2Vec removes some of the long (more than 250 letters) SMILES during preprocessing and conducts one-hot coding on the remaining SMILES, converting each SMILES into a vector of length 26. Based on this pretreatment method, the chemical structure of the drug is pretreated, as shown in Figure 2.

All the SMILES stored in DrugBank are converted into a word bag with 251 elements. Then, one-hot encoding is used to transform them into 251 dimensional vectors. Finally, PCA is used to reduce the 251-dimensional SMILES vectors to a specific dimension. Thus, we obtain a vector of lower dimension used to represent the structural feature of a drug.

### 2.2. Drug Topological Features

Construction of KG. The data from two databases are used to construct KG, which are then used to obtain the topological features of the drugs. The Kyoto Encyclopedia of Genes and Genomes (KEGG) [21] is a database resource for understanding advanced functions and utilities of biological systems from molecular-level information. There are multiple sub-databases under KEGG. Wang et al. [22] constructed a large, high-quality heterogeneous map linking the Patient, Disease, and Drug (PDD) into an Electronic Medical Record (EMR). The PDD database extracts key medical entities from MIMIC-III (Medical Information Mart for Intensive Care III) [23] and links them to current biomedical knowledge graphs (including ICD-9 Ontology and DrugBank). PDD diagrams are accessible on the web through SPARQL endpoints and provide information for medical research and treatment recommendations.

**Figure 3:**
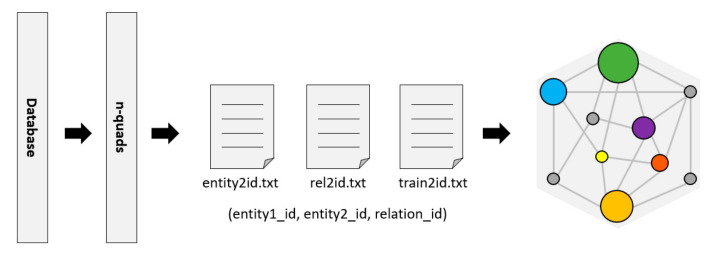
KG construction.

RDF (Resource Description Framework) [24] is a resource description language commonly used as a representation of the KG. The Bio2RDF project [25] provides tools to convert data to n-quads or other formats of RDF. Then, the RDFlib library is used to parse these n-quads data and divide them into triples (entity, relationship, and entity) in a format that is convenient for KG to generate embedded features, as shown in Figure 3.

Here, we introduce a metric named density to evaluate the KG. Density is used to describe the connection’s density between nodes in a graph/network. For a graph *G* with *L* edges and *N* nodes, the density calculation formula is shown in (1):

(1) $$d\left(G\right)=\frac{2L}{N\left(N-1\right)}$$

The density of the graph has a certain influence on the results of graph-based research and machine learning. This will be discussed in subsequent experiments.

We construct two KGs by KEGG and PDD, respectively. The corresponding data is shown in Table 1.

It can be seen from the table that there are more types of drugs in the KEGG data set but the graph itself is relatively sparse and the proportion of drugs with structure records is relatively lower. The PDD dataset has fewer drug types but the graph is denser and the proportion of drugs with structure records is higher.

**Figure 4:**
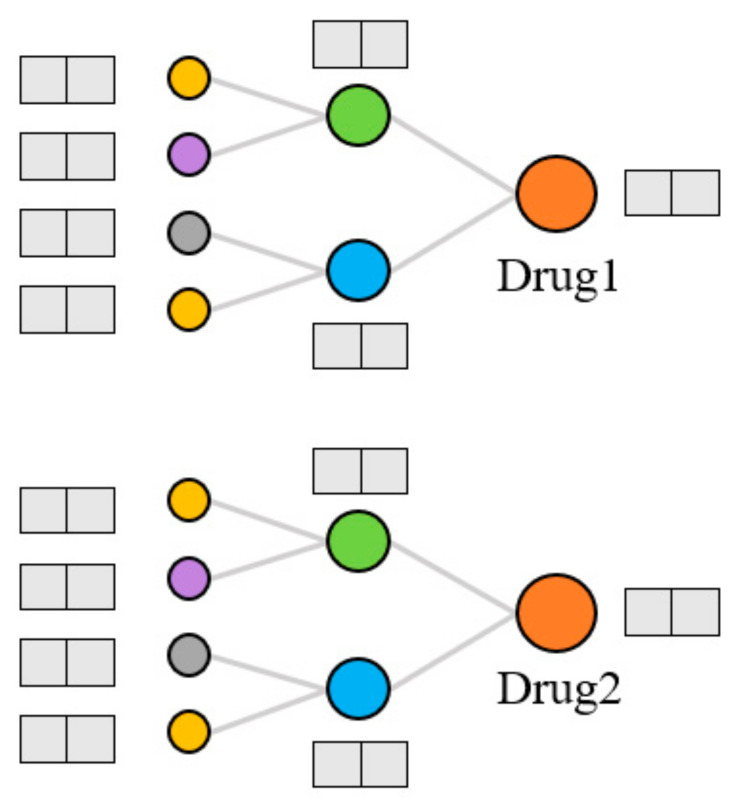
Extraction of topological features.

Extraction of topological features. Generally, the models that use KG to predict DDIs can only capture data information in a small range. The KGNN [9] model was proposed to expand the receptive field, obtain the rich entity information in the KG, and explore the potential correlation between drugs and other entities. It extracts the higher-order structure and semantic relations of drugs by GNN and learns the representation of drugs and their neighborhoods from the KG. We used the KGNN model to calculate the topological features of drugs on the KG, as shown in Figure 4. For each entity, the model extracts several entities from the domain of the entity and aggregates the information of these entities to form the topological feature representation of the entity. There are three kinds of entity aggregation methods: sum aggregation is a superposition operation, concatenate is a concatenate operation, and neighbor only aggregates information from the neighborhood but not the node itself. These three aggregation methods are abbreviated as sum, concat, and neigh, respectively.

### 2.3. Drug–Drug Interaction Prediction

**Figure 5:**
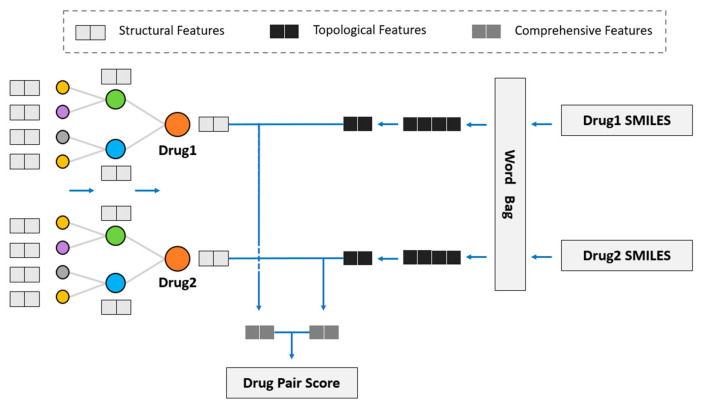
SmileGNN model.

We considered using GNN to obtain the drug topological features on the KG and fuse drug structural features into the model to study the influence of drug structural features on DDI prediction. Hence, we propose the novel model of SmileGNN, as shown in Figure 5.

For drugs, the structural features are the vectors that we obtain from SMILES structural data using the method mentioned in Section 2.1 to indicate its structural characteristics. The topological features refer to the vectors which record relationships between the drug and other molecules in KG learned through GNN. The KG is established using the method in Section 2.2. Comprehensive features refer to the vectors obtained by aggregating the structural features and topological features of the drug (see Section 3.4 for detailed aggregation methods) to represent the drug.

The algorithm can be summarized as follows. The method SMILES2Vec mentioned in Section 2.1 is used to calculate the structural features by using the data of SMILES. The KGNN model is retained to calculate the drug topological features, in which the graph neural network (GNN) is used to aggregate the entity information of the receptive field within two hops of the entity to obtain the drug topological features. Then, the two features of the drug are aggregated to obtain a comprehensive drug feature, including drug topological features and drug structural features. Two algorithms are specifically designed to aggregate drug structural features and drug topological features. See Section 3.4 for detailed algorithms and a comparative analysis.

After obtaining the comprehensive features of the two drugs, we dotted and summed the features. The drug pair score was obtained through sigmoid function and hence was distributed in the interval of (0, 1). It is classified as the presence of DDI if the interaction value exceeds 0.5 and otherwise as the absence of DDI.

It should be noted that the positive and negative samples in the experiment are not the results of manual labeling but rather come from the existing data in the database. The negative samples in this article are considered to be no DDI between the two drugs but the possibility of existing DDI between the two drugs is not excluded. We can only say it has not been clinically verified, thus it has not been recorded in the database.

This model uses the dichotomous cross-loss entropy as the loss function and its calculation formula is shown in (2):

(2) $$Loss=\sum_{\left(i,j\right)\in Y\left(i,j\in N_{d},j\neq i\right)}-y_{i,j}log\hat{y_{i,j\;}}-\left(1-y_{i,j}\right)\log\left(1-\hat{y_{i,j\;}}\right)$$

where $\hat{y_{i,j\;}}$ represents the predicted value, $y_{i,j}$ represents the true value of drug pairs in the data set, and *Y* represents the set of all drug pairs.

## 3. Experiment

### 3.1. Experimental Settings

In this paper, the prediction of DDI is considered as a binary task. It does not necessarily predict the specific type of DDI or what side effects the DDI may cause but only judges whether there is a possible DDI between the drug pair.

Metrics. ACC (Accuracy) and AUC (Area Under Curve) are used as the main evaluation metrics for a series of models. In some comparative experiments, the F1-Score is also used as a metric.

Settings. The experiment was conducted on two datasets, namely KEGG and PDD. Section 2.2 shows the construction and data features of the respective dataset. For the two datasets, a parameter combination that achieves the highest AUC value was adopted through parameter tuning based on grid search. The final parameters to be used are shown in Table 2.

Baselines. In addition to KGNN, two classic models, namely DeepDDI and Decagon, were compared with the new model proposed in this paper. See Section 1 for a detailed introduction of the models.

DeepDDI [4]: The DeepDDI model is based on the drug structural feature method and is the first to use a deep neural network to predict DDI. The model was put forward in 2017 and established the Gold Standard Database (Gold Standard Database) of DDIs. DeepDDI is considered a benchmark among structural feature methods.

Decagon [7]: The Decagon model is the first model using a graph neural network among graph-based methods. This model was proposed in 2018 and is a model with great influence among graph-based methods in recent years.

KGNN [9]: The usage of KG and GNN to predict DDI can mine the potential correlations between drugs and other entities.

### 3.2. Results and Analysis

The experimental results of these models were compared and analyzed, as shown in Table 3.

SmileGNN achieved the best performance among all the models. Compared to the classic DeepDDI and Decagon models, there was a 5.3% and 8.0% improvement in AUC values, respectively. Compared with the KGNN model using drug topological features alone, it also has a certain performance improvement.

Although both the DeepDDI model and Decagon model are the pioneer models in the field of DDI prediction, the model designs still need to be improved and their prediction performance is relatively poor. Though both are graph-based methods, the Decagon model only uses the topological features of the drug, while the KGNN model considers the topological features of both the current node and the nodes in the neighborhood of the drug within a certain range, thus more information can be learned from the graph. This results in an improved performance compared with the Decagon model. The new model SmileGNN proposed in this paper combines the topological features and structural features of the drug, and performs better in terms of the DDI prediction than the Decagon and KGNN models that extract topological features alone or the DeepDDI model that uses structural features alone.

The SmileGNN model retains the method of the KGNN model in learning drug topological features and has an excellent performance. However, in terms of the learning of drug structural features, the model proposed in this paper deals with SMILES in a relatively independent and rough way. Future research can further optimize the feature expression algorithm of drug structural features to improve the prediction ability of the model.

### 3.3. Ablation Study

SmileGNN adds the use of drug structural features to KGNN and integrates multi-source information to predict new DDIs. An ablation experiment was conducted to compare and analyze the influence of the new drug structural features with the performance of the original KGNN model [9].

Experiments were carried out in the KEGG and PDD datasets on the three drug topological feature aggregation types of sum, concat, and neigh. The aggregator mentioned here was used to combine the feature of the current node and the nodes in the neighborhood of the drug within a certain range. The experimental results are shown in Table 4.

For both the KEGG and PDD datasets, the performance of SmileGNN, which additionally uses drug structural features, was better than that of KGNN in all the three kinds of aggregation methods of drug topological features. Consistent with the KGNN model, SmileGNN achieved the best effect when using concat for obtaining drug topological features, with the AUC value reaching 0.9521 and 0.9642 in the KEGG and PDD dataset, respectively. This proves that the newly added drug structural features can steadily improve the performance of the model.

Table 4 reveals that the performance of both the KGNN and SmileGNN models on the PDD dataset is better than that using the KEGG dataset. As for the improvement of model performance after adding SMILES, it obtained the same degree of improvement on the PDD dataset, with about a 1% improvement in the ACC, AUC, and F1 value.

Based on the comparison of the KEGG and PDD datasets in Section 2.2, the following conclusions can be basically drawn:

1. On the denser graph, the drug topology information learned from the model is richer and can better represent the drug topological features.
2. In PDD data, there is a higher proportion of drugs that have corresponding drug structures. Even with a higher start, the performance of using the PDD dataset still improved by about 1% by adding structural features. Thus, drug structural features have a great positive influence on the model, which is positive.

Due to the limitations of the dataset, that is, the fact that drug pairs classified as without DDIs may actually have DDIs, the predicted results of the model cannot be infinitely close to 1 and the excellent performance obtained in both the training and cross-validation does not explain everything. In Section 4, special attention is paid to drug pairs that are classified “incorrectly”, i.e., those that the datasets recorded as non-DDIs but that the model predicted as DDIs.

### 3.4. Case Study

- Influence of the drug feature aggregation method

Referring to the ways that KGNN was designed to aggregate the topological features of multiple nodes together, methods sum and concat are designed to aggregate the structural features and topological features of drugs together by corresponding superposition operation and concatenate operation.

We have two matrices as input: drug topological feature matrix A, whose shape is ${\mathrm{BatchSize}\;*\;\mathrm{EmbedDimension}}_{A}$, and drug structural feature matrix B, whose shape is ${\mathrm{BatchSize}\;*\;\mathrm{EmbedDimension}}_{B}$. For the sum method, the weight matrix W of the shape ${\mathrm{EmbedDimension}}_{A}{\;*\;\mathrm{EmbedDimension}}_{A}$ is designed and the bias vector is b. Notice that the matrices A and B have to have the same shape. Output is shown in Formula (3). For the concat method, the weight matrix W of the shape $\left({\mathrm{EmbedDimension}}_{A\;}+{\;\mathrm{EmbedDimension}}_{B}\right){\;*\;\mathrm{EmbedDimension}}_{A}$ is designed and the bias vector is b. Output is shown in Formula (4).

(3) $$\tan h(\left[\;A+B\right]\;*\;W+b)$$

(4) $$\tan h(\left[\;A+B\;\right]\;*\;W+b)$$

For the PDD dataset, when other parameters are unchanged, the drug topological feature dimension is set as 64 dimensions. So is the drug structural feature dimension. The two aggregation methods were used to obtain drug comprehensive features and the other parameters were consistent. The experimental results are shown in Table 5.

As can be seen from Table 5, when sum and concat are used to aggregate drug topological features and drug structural features, the performance of the sum method is slightly better than that of the concat method, but the difference is not significant. In view of the fact that the concat method is more flexible and has no requirement on the feature dimension, subsequent experiments all adopted the concat method.

- Influence of the drug structural feature dimension

To measure the influence of the drug structural feature dimension on the result of the model training and to study the loss of the PCA dimension reduction method, we conducted the following experiment. The concat method was used to connect drugs’ topological features and structural features using the PDD dataset. Set the PCA dimension reduction of the drug structural feature dimension as 32 d, 64 d, and 96 d. Other parameters remain the same.

**Figure 6:**
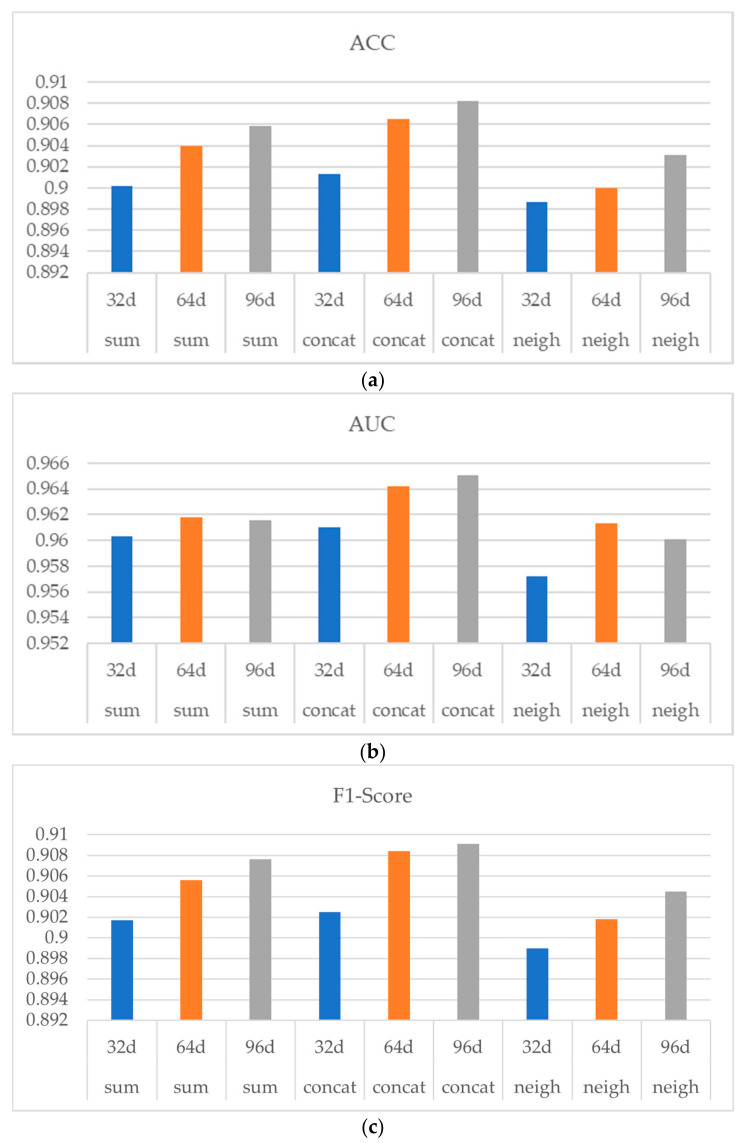
Influence of the drug structure characteristic dimension on model performance. (**a**) Influence of different dimension on ACC. ACC increases with the increasement of drug structural feature dimension. (**b**) Influence of different dimension on AUC. AUC reaches the highest when drug structural feature dimension is 64. (**c**) Influence of different dimension on F1-Score. F1-Score increases with the increasement of drug structural feature dimension.

Among them, three methods of sum, concat, and neigh are used to obtain drug topological features in order to observe whether the influence of drug structural feature dimensions is stable and consistent. Figure 6 shows the experiment result, in which (a) (b) and (c) indicates ACC, AUC and F1-Score respectively. As shown in Figure 6, with the increasing of the drug structure feature dimension from 32 d to 64 d, the performances of the three aggregators were all improved, indicating a stable and consistent influence of the drug structural feature dimension on the model performance. Note that when the drug structural dimension was increased from 64 d to 96 d, the performance of the model was not significantly improved.

In conclusion, when PCA is used to reduce the dimension of drug structural features, the effect of the dimension reduction is better and the information loss is smaller in the process of decreasing from 251 d to 64 d. When the dimension is further reduced, the representation of the drug structural features may be greatly lost and the performance of the final model will be affected. Considering when using 64 d drug structural features, the model has already had a relatively good performance, while the use of a higher dimension of drug structural features will occupy more computing resources and storage space, and the performance improvement is not obvious, thus the experiments uniformly used 64 d drug structural features.

## 4. Discussion

Instead of sending the score of the drug pairs into the threshold category of 0.5, the drug pairs with a score over 0.9 were directly printed and ranked from highest to lowest. To obtain a better result, we used the PDD dataset to have drug pairs classified as DDIs and to eliminate pairs which were recorded with DDIs in PDD. We then obtained the highest score of the top ten new predictions of DDIs and sent the results to the latest DrugBank database query. The ones that were recorded as DDI in DrugBank were marked as 1 and otherwise marked as 0, as shown in Table 6. Table 7 shows the corresponding drug names to the DB numbers from DrugBank.

The PDD dataset was updated to version 1.3 and uploaded in October 2018. The DDIs in the PDD dataset were extracted from version 5.1.1 of DrugBank, which was uploaded in July 2018. The latest DrugBank database is version 5.1.8, uploaded in January 2021. Thus, there is a 2.5-year gap during which many new DDIs were discovered and verified.

It can be seen that the five new DDIs shown in Table 6 have been clinically verified and included in the DrugBank database in the recent two years, while the remaining five DDIs have not been experimentally verified yet. The model proposed in this paper is reliable for the prediction of novel DDIs and the experimental results are of great supporting significance for clinical trials of novel DDIs.

In the following paragraphs, two drug pairs were studied separately and the influence of the drug structural features and drug topological features on drug pair interaction prediction is discussed. It can be seen that drug pairs [DB00437, DB00959] and [DB00437, DB00633] have high scores above 0.99 and both contain drug DB00437.

According to the SSP calculated in DeepDDI [4], it is known that the structural similarity between drug DB00959 and drug DB00633 is only about 35.19%, which is not high. However, only 72% of the drugs in the PDD dataset have SMILES data. Thus, for about 48% of the drug pairs, their structural similarity cannot be directly calculated. In the context of sparse data, a 35.19% similarity also has a great impact on the results.

In the drug targeting data, it was found that both drug DB00959 and drug DB00633 acted on the Cytochromes P450 group protein enzymes. Due to the similar pathway of action, the model was more inclined to believe that drug DB00959 and drug DB00437 also have DDIs. The DDI records in the DrugBank database show that the adverse drug event of drug combination [DB00437, DB00633] is due to competition for the excretory pathway of the kidney [26]. Based on the relevant information in literature and on a series of databases, it is believed that the interaction mechanism of this drug pair is not obviously related to the protein enzymes of the Cytochromes P450 group [27,28].

Through the study of this example, it is realized that SmileGNN can make good use of the known drug structural information and drug topological information to predict DDIs. However, due to limitations caused by the insufficient information of the drug structure and the relatively blind and random nature embedded in the learning of the topological information in KG, the SmileGNN model still has much room for improvement in learning drug features. 

## 5. Conclusions

In this paper, the new model SmileGNN (model based on SMILES and the graph neural network) was proposed to predict drug–drug interactions by comprehensively using drug structural features and drug topological features. We implemented the proposed method and conducted experimental comparisons on two datasets. The results verified that SmileGNN has better performance than the classic models and KGNN. Based on the latest database, SmileGNN’s prediction results are also credible.

## Figures and Tables

**Table 1:** Comparison of KEGG KG and PDD KG.

	KEGG	PDD
Number of drugs	11,174	1495
The proportion of drugs with structural records	13.96%	72.37%
The density of the graph	4.300 × 10^−5^	8.571 × 10^−4^
Number of positive samples	56,983	36,768
Drug-drug interaction subgraph density	9.128 × 10^−4^	3.292 × 10^−2^

**Table 2:** Experimental parameters.

	KEGG	PDD
Batch size	2048	1024
Learning rate	2 × 10^−^^2^	1 × 10^−^^2^
GNN embed dimension	32	64

**Table 3:** Comparative analysis of the new model and several classical models.

Model	The Data Source	ACC	AUC
DeepDDI	KEGG	0.8217	0.8987
Decagon	STITCH, etc.	--	0.8720
KGNN	KEGG	0.8834	0.9422
SmileGNN	KEGG	0.8936	0.9521

**Table 4:** Comparison of the performance of SmileGNN and KGNN on datasets.

Dataset	Model	Aggregator Type	Average Accuracy	Average AUC	Average F1-Score
KEGG	KGNN	sum	0.8801	0.9390	0.8851
		concat	0.8834	0.9422	0.8881
		neigh	0.8642	0.9267	0.8690
		Average	0.8759	0.9360	0.8807
	SmileGNN	sum	0.8888	0.9467	0.8943
		concat	0.8936	0.9521	0.8957
		neigh	0.8744	0.9329	0.8788
		Average	0.8856	0.9439	0.8896
PDD	KGNN	sum	0.8920	0.9542	0.8947
		concat	0.8970	0.9576	0.8995
		neigh	0.8896	0.9518	0.8919
		Average	0.8929	0.9545	0.8954
	SmileGNN	sum	0.9040	0.9618	0.9056
		concat	0.9065	0.9642	0.9084
		neigh	0.9000	0.9613	0.9018
		Average	0.9035	0.9624	0.9053

**Table 5:** Different aggregation methods on the PDD dataset.

	ACC	AUC	F1-Score
sum	0.9095	0.9647	0.9070
concat	0.9056	0.9618	0.9040

**Table 6:** New DDIs.

Drug1	Drug2	Score	Whether You Can Query DDI in DrugBank
DB00437	DB09322	0.999964	0
DB00450	DB00768	0.999917	0
DB00437	DB00959	0.999854	0
DB00660	DB01656	0.999831	1
DB00722	DB01039	0.999817	1
DB00437	DB00633	0.999764	1
DB00346	DB01173	0.999618	1
DB04908	DB05521	0.999571	1
DB00475	DB00820	0.999542	0
DB00040	DB00564	0.999236	0

**Table 7:** The corresponding drug names of drugs in new DDIs.

Drug1	Drug1 Name	Drug2	Drug2 Name
DB00437	Allopurinol	DB09322	Zinc sulfate
DB00450	Droperidol	DB00768	Olopatadine
DB00437	Allopurinol	DB00959	Methylprednisolone
DB00660	Metaxalone	DB01656	Roflumilast
DB00722	Lisinopril	DB01039	Fenofibrate
DB00437	Allopurinol	DB00633	Dexmedetomidine
DB00346	Alfuzosin	DB01173	Orphenadrine
DB04908	Flibanserin	DB05521	Telaprevir
DB00475	Chlordiazepoxide	DB00820	Tadalafil
DB00040	Glucagon	DB00564	Carbamazepine

## Data Availability

All the codes are available at: https://github.com/AshleyHan/SmileGNN.
